# G-CSF inhibits LFA-1-mediated CD4^+^ T cell functions by inhibiting Lck and ZAP-70

**DOI:** 10.18632/oncotarget.18194

**Published:** 2017-05-25

**Authors:** Shasha Zhao, Zhenyang Gu, Li Wang, Lixun Guan, Feiyan Wang, Nan Yang, Lan Luo, Zhe Gao, Yingwei Song, Lili Wang, Daihong Liu, Chunji Gao

**Affiliations:** ^1^ Department of Hematology, Chinese PLA General Hospital, Beijing 100853, China; ^2^ Medical School, Nankai University, Tianjin 300071, China; ^3^ Department of Hematology and Oncology, Laoshan Branch, No. 401 Hospital of Chinese PLA, Qingdao 266101, China; ^4^ Department of Blood Transfusion, Chinese PLA General Hospital, Beijing 100853, China

**Keywords:** granulocyte colony-stimulating factor, CD4^+^ T cells, lymphocyte function-associated antigen-1, Lck, ZAP-70

## Abstract

In this study, we showed that G-CSF mobilization increased the frequency of T cells, specifically CD3^+^CD4^+^ T cells. G-CSF mobilization decreased the secretion of inflammatory cytokines of CD4^+^ T cells through the LFA-1/ICAM-1 signaling pathway, whereas it did not alter the TH1/TH2 ratio. We found that G-CSF mobilization inhibited LFA-1-mediated CD4^+^ T cell polarization and motility. *In vitro*, G-CSF stimulation also attenuated the polarization and adhesiveness of CD4^+^ T cells through the LFA-1/ICAM-1 interaction. Further investigation revealed that G-CSF mobilization suppressed LFA-1 signaling by down-regulating Lck and ZAP-70 expression in CD4^+^ T cells, similar results was also confirmed by *in-vitro* studies. These findings suggested that G-CSF directly suppressed LFA-1-mediated CD4^+^ T cell functions through the down-regulation of Lck and ZAP-70. The immunosuppressive effect of G-CSF mobilization deepened our understanding about peripheral blood hematopoietic stem cell transplantation. LFA-1/ICMA-1 pathway may become a potential target for graft-versus-host disease prophylaxis.

## INTRODUCTION

Granulocyte colony-stimulating factor (G-CSF), which can mobilize hematopoietic stem cells (HSCs) from the bone marrow to the peripheral blood, is widely used in the field of transplantation. G-CSF is a multiple-functional glycoprotein which plays an important role in regulating adaptive immune responses [1, 2]. G-CSF has both direct and indirect effects on immune cells, including monocytes, granulocytes, T cells and dendritic cells. Moreover, G-CSF alters the expression of cytokines, metalloproteinases and adhesion molecules. These various soluble factors may contribute to the effects on immune cells induced by G-CSF [3–5].

Graft-versus-host disease (GVHD), induced by alloreactive T cells, is a major complication of allogeneic hematopoietic stem cell transplantation (allo-HSCT) [6, 7]. Thus, suppressing the function of alloreactive T cells has been a major strategy to reduce GVHD [8, 9]. Based on their cytokine expression pattern, CD4^+^ T cells can be divided into TH1 and Th2 cells. TH1 cells can secrete interleukin (IL)-2, tumor necrosis factor (TNF)-α and interferon (IFN)-γ and play a major pro-inflammatory role in cellular immunity against intracellular pathogens, whereas TH2 cells, characterized by the production of IL-4, IL-5 and IL-10, paly a predominant role in humoral immunity against extracellular parasites [10]. The ratio of TH1/TH2 cells varied with different pathophysiological conditions, involving in the progression of or protection against some infectious and autoimmune conditions [11, 12].

Lymphocyte function-associated antigen-1 (LFA-1) is an integrin expressed on T cells, B cells, NK cells, and neutrophils [13]. Activated LFA-1 interacts with its ligand, intracellular adhesive molecules 1 (ICAM-1), which is inducibly expressed on high endothelial venules and other types of cells during inflammation. The LFA-1/ICAM-1 interaction plays a key important role in the adhesion, locomotion and migration of T cells [14, 15], thus increasing the avidity of the T cell/APC interaction at the level of the immunological synapse [16]. Therefore, it is crucial for the activation of T cells [17]. Additionally, LFA-1/ICMA-1 signaling pathway also participates in many intracellular activities, like the remodeling of cytoskeletal systems and activities of many proteins such as signaling proteins, adaptor proteins, enzymes, and transcription factors [18–22].

We have previously found that G-CSF mobilization can inhibit the proliferation and activation of CD4^+^ T cells through the conformation of LFA-1 [23]. These findings prompted us to consider the possibility of a crucial cross-talk between G-CSF and LFA-1 signaling in the function of CD4^+^ T cells. In this study, we found that G-CSF decreased the expression of Lck and ZAP-70 in the LFA-1 signaling pathway, which ultimately inhibited CD4^+^ T cell functions.

## RESULTS

### G-CSF mobilization increased the frequency of T cells, specifically CD3^+^CD4^+^ T cells

**Figure 1:**
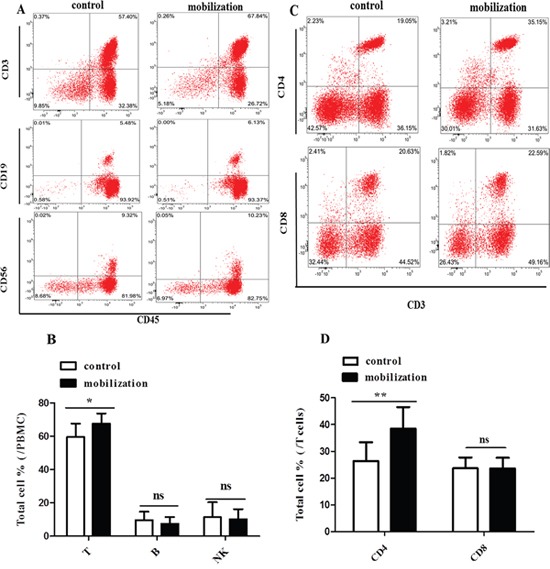
Effect of G-CSF mobilization on lymphocyte and T cell subtypes **(A)** PBMCs were collected from healthy volunteers and G-CSF-mobilized donors. Lymphocyte subpopulations were stained with corresponding immunophenotypic markers and analyzed by flow cytometry. **(B)** The frequency of lymphocyte subsets is summarized in the graph. **(C)** T cell subpopulations were stained with corresponding fluorescents labeled antibodies and analyzed by flow cytometry. **(D)** The frequency of T cell subsets is summarized in the graph. Representative flow cytometry images are shown. The data are presented as the mean ± SD (n = 10 per group). ns: no significant difference; * *P* < 0.05; **P < 0.01.

Clinical results have shown that the incidence and severity of acute GVHD is comparable between peripheral blood stem cell (PBSC) transplantation and bone marrow transplantation (BMT) [24–26], even though PBSC grafts contain approximately 10-fold more T cells than conventional BM grafts [27–29]. A possible reason is that G-CSF, which is used for mobilizing PBSC, alters the functional activities of donor T cells. In this study, we first analyzed the effects of G-CSF mobilization on lymphocyte and T cell subsets. Peripheral blood mononuclear cells (PBMCs) were obtained from healthy volunteers (control group) and G-CSF mobilized HSC donors (G-CSF mobilization group) with informed consent. Then, PBMCs were stained with corresponding fluorescents labeled antibodies and analyzed by flow cytometry. G-CSF mobilization dramatically increased the frequency of T cells (67.63 % vs. 59.54%, *P*=0.021) but not B cells and NK cells (Figure 1A and 1B). T cell subsets analysis showed that the percentage of CD3^+^CD4^+^ T cells was significantly higher in G-CSF mobilized group than in the control group (38.51% vs. 26.41%, *P*=0.003). However, the frequency of CD3^+^CD8^+^ T cells was not affected (23.74 % vs. 23.62%, *P*=0.946) (Figure 1C and 1D). These data suggested that G-CSF mobilization increased the frequency of T cells, specifically CD3^+^CD4^+^ T cells. This finding prompted us to further investigate whether and how G-CSF mobilization influences the functions of CD4^+^ T cells.

### G-CSF mobilization inhibited the release of inflammatory cytokines from CD4^+^ T cells through the LFA-1/ICAM-1 interaction

**Figure 2:**
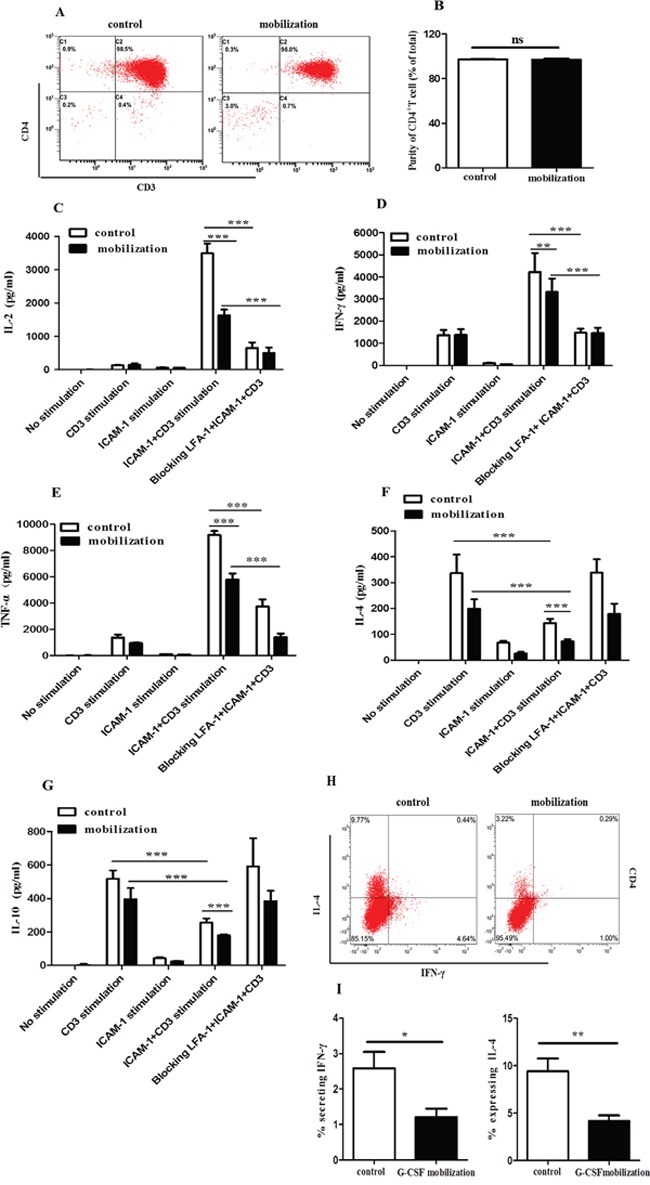
G-CSF mobilization inhibited the secretion of inflammatory cytokines from CD4+ T cells CD4^+^ T cells were purified from the peripheral blood of healthy volunteers and G-CSF-mobilized donors. CD4^+^ T cells were isolated using magnetic beads conjugated to a human CD4 antibody. **(A)** The purity of the isolated cells was then analyzed by flow cytometry. **(B)** The purified cells from the two groups were quantified (n=15 per group). Unstimulated, and ICAM-1-stimulated CD4^+^ T cells from G-CSF-mobilized donors and healthy volunteers were cultured with or without anti-CD3 or ICAM-1 plus anti-CD3 for an additional 72 h. To inhibit LFA-1-mediated signalings, the cells were treated with anti-LFA-1 blocking Ab. At the end of the incubation period, the culture supernatants were harvested, and the secreted levels of IL-2 **(C)**, IFN-γ **(D)**, TNF-α **(E)**, IL-4 **(F)** and IL-10 **(G)** were analyzed by the ProcartaPlex™ Multiplex Immunoassay (n=13 per group). **(H)** IFN-γ and IL-4 production by CD4^+^ T cells, evaluated by intracellular IFN-γ and IL-4 staining. **(I)** The percentage of IFN-γ and IL-4 expression is summarized in the graph (n=8 per group). The data are shown as the mean ± SD. ns: no significant difference; * *P* < 0.05; ** *P* < 0.01; *** *P* < 0.001.

We previously observed that G-CSF decreased CD4^+^ T cell activation and proliferation by changing the conformation of LFA-1 [23]. Verma and colleagues demonstrated that LFA-1/ICAM-1 ligation could induce the polarization of T cells to TH1 cells [30]. We then investigated whether G-CSF mobilization influences CD4^+^ T cells through ICAM-1/LFA-1 interaction. CD4^+^ T cells were isolated from healthy volunteers (control group) and G-CSF mobilized HSC donors (G-CSF-mobilization group). The purity of the CD4^+^ T cells was greater than 96% in the two groups (Figure 2A). There was no difference in the purity of CD4^+^ T cells between the control group and the G-CSF-mobilization group (Figure 2B). The culture supernatants were collected after stimulation of purified CD4^+^ T cells in different conditions for 72h, and the levels of the cytokines, including IL-2, IL-4, IL-10, IFN-γ and TNF-α, were detected. The results showed that incubation of CD4^+^ T cells with either ICAM-1 or anti-CD3 increased the IL-2 level (Figure 2C). This increase in IL-2 secretion was also observedwhen the cells were stimulated withboth ICAM-1 and anti-CD3. The increase of IL-2 was abrogated when anti-LFA-1 blocking antibody was used (Figure 2C). Compared with the control group, G-CSF mobilization inhibited IL-2 production by > 50% when the cells were stimulated with both ICAM-1 and anti-CD3 (Figure 2C). Furthermore, LFA-1/ICAM-1 signaling in CD4^+^ T cells increased the anti-CD3-mediated production of IFN-γ and TNF-α, which was abrogated in thepresence of anti-LFA-1 blocking antibody (Figure 2D and 2E). G-CSF mobilization also significantly inhibited the release of IFN-γ and TNF-α when the cells were stimulated with both ICAM-1 and anti-CD3 (Figure 2D and 2E). In contrast, LFA-1/ICAM-1 stimulation in CD4^+^ T cells significantly decreased anti-CD3-mediated secretion of IL-4 (Figure 2F) and IL-10 (Figure 2G). In addition, the ICAM-1- and anti-CD3-stimulated secretion of IL-4 and IL-10 in the control group showed~2-fold than the G-CSF-mobilization group (Figure 2F and 2G). We further analyzed the cellular expression of TH1/TH2 cytokines in LFA-1/ICAM-1- and anti-CD3-stimulated CD4^+^ T cells using flow cytometry (Figure 2H). CD4^+^ T cells from the G-CSF–mobilization group exhibited significant different in cytokine expression. In comparison to the control group, G-CSF mobilization decreased IFN-γ and IL-4 production by more than 50% (Figure 2I), indicating that G-CSF mobilization suppressed the percentage of TH1 and TH2 cells. However, there was no statistically significant difference in the TH1/TH2 ratio between the G-CSF mobilization group and the control group (data not shown). Collectively, these data supported the critical involvement of LFA-1 signaling in CD4^+^ T cell cytokine secretion and suggested that G-CSF mobilization decreased the release of inflammatory cytokines from CD4^+^ T cells through the LFA-1/ICAM-1 interaction but did not alter the balance of the TH1/TH2 subsets.

### G-CSF mobilization decreased the polarization and migration of CD4^+^ T cells

**Figure 3:**
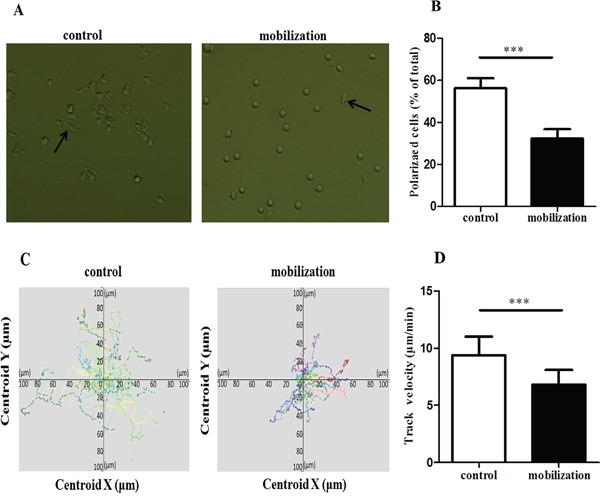
G-CSF reduced the polarization and migration of CD4+ T cells **(A** and **B)** Control and mobilized CD4^+^ T cells were added to anti-CD3- and ICAM-1-coated 96-well plates. After incubation at 37°C for 60 minutes, the cells were analyzed by fluorescence microscopy. Arrows indicate polarized T cells with a clear uropod. (B) Cells prepared as in A were quantified. **(C** and **D)** The two groups of CD4^+^ T cells were loaded on a coated surface, and cell movements were recorded by confocal microscopy. The cell tracks and motility were analyzed by the Velocity software. Tracks of individual cells are presented with the same point of origin. All data are represented as the mean ± SD (n=15 per group). *** *P* < 0.001.

Given the crucial role of LFA-1/ICAM-1 in T cell attachment, polarization and migration, we examined whether G-CSF mobilization could affect LFA-1-mediated CD4^+^ T cell polarization and migration. Purified CD4^+^ T cells were stimulated in 96-well plates coated with anti-CD3 and ICAM-1 and then visualized by fluorescence microscopy after 60 minutes of stimulation. Polarization morphology was detected in the control group and the G-CSF mobilization group (Figure 3A). However, the percentage of polarized cells was significantly lower in the mobilized group than in the control group (32.37% vs. 56.32%, *P*<0.0001) (Figure 3B). Moreover, the motility and velocity of the mobilized CD4^+^ T cells were significantly lower than that of the control group (average speed of 6.82 vs. 9.39 μm/min, *P*<0.0001) (Figure 3C and 3D). Taken together, these results showed that G-CSF mobilization can decrease the polarization and migration of CD4^+^ T cells.

### G-CSF stimulation attenuated the polarization and adhesiveness of CD4^+^ T cells *in vitro*

**Figure 4:**
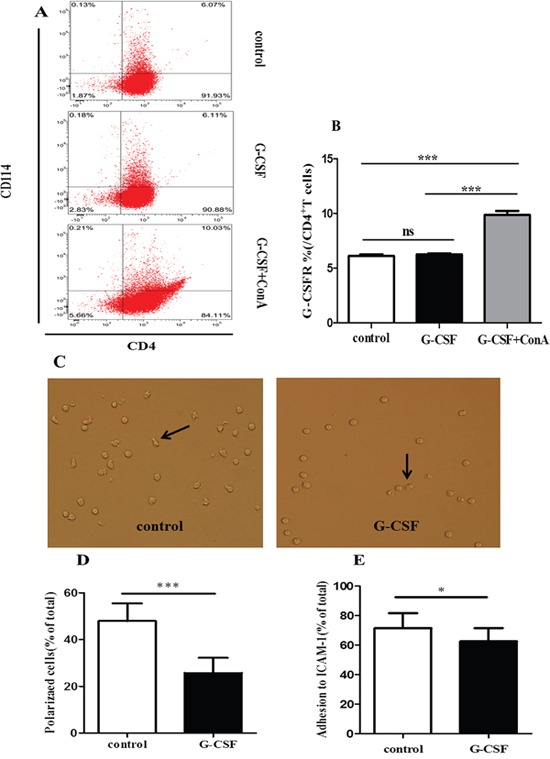
G-CSF attenuated the polarization and adhesion of CD4+ T cells *in vitro* Purified CD4^+^ T cells were added to 24-well plates and incubated in RPMI alone or in RPMI containing G-CSF or G-CSF plus ConA for 24 h. **(A)** The expression of G-CSFR on CD4^+^ T cells, evaluated by CD114 staining. **(B)** The percentage of G-CSFR is summarized in the graph (n=5 per group). After incubation 24h, G-CSF stimulated or left un-stimulated CD4^+^ T cells were transferred to 96-well plates coated with ICAM-1 and anti-CD3. **(C)** Cell morphology was examined by fluorescence microscopy at 60 minutes. Arrows indicate polarized T cells with a clear uropod. **(D)** Cells prepared as in C were quantified. **(E)** Unbound cells were washed off, and the adherent cells were quantified using a CCK-8 assay kit. The data are represented as the mean ± SD (n=15 per group). * *P*< 0.05; *** *P* < 0.001.

G-CSF directly affects lymphocytes functions through the G-CSFR [31, 32]. We first analyzed the G-CSFR levels on CD4^+^ T cells. Purified CD4^+^ T cells were un-stimulated (control group) or stimulated with G-CSF (G-CSF group) or G-CSF plus ConA (G-CSF+ConA group) *in vitro*. There was an approximately 2-fold increase in levels of G-CSFR expression on CD4^+^ T cells in G-CSF+ConA group compared with control and G-CSF group (10%, 6.13%, 6.26%, respcetively) (Figure 4A and 4B). This result indicated that ConA could induce G-CSFR expression in CD4^+^ T cells. We next determined whether the observed decrease in polarization was a direct effect of G-CSF on CD4^+^ T cells. Although both unstimulated and G-CSF-stimulated CD4^+^ T cells exhibited polarization morphology (Figure 4C), the percentage of polarized cells was significantly lower in the G-CSF group than in the control group (25.70% vs. 47.90%, *P*<0.0001) (Figure 4D). Moreover, the adhesiveness of the CD4^+^ T cells in the G-CSF group was also weaker than that of the control group (62.62% vs. 71.61%, *P*=0.015) (Figure 4E). These data suggested that G-CSF could directly inhibit the polarization and adhesiveness of CD4^+^ T cells.

### G-CSF mobilization decreased Lck and ZAP-70 expression on CD4^+^ T cells

**Figure 5:**
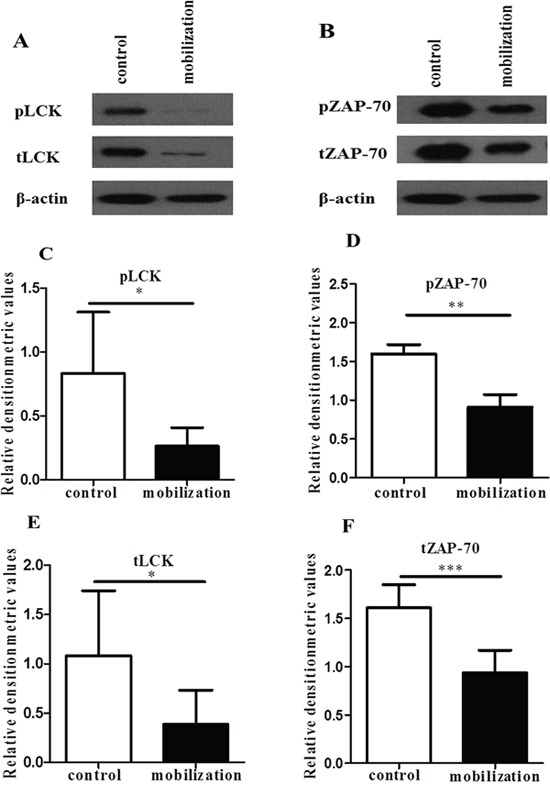
G-CSF decreased the expression of Lck and ZAP-70 Purified control and mobilized CD4^+^ T cells were added to anti-CD3- and ICAM-1-coated 6-well plates. After an incubation at 37°C for 1 h. **(A** and **B)** The cell lysates were analyzed by western blot to detect the expression of Lck, ZAP-70 and phosphorylated Lck and ZAP-70, which were normalized to actin. **(C-F)** Relative densitometric analysis of the individual protein bands. The data are shown as the mean ± SD (n= 6 per group). * *P* < 0.05; ** *P* < 0.01; *** *P* < 0.001.

Previous studies have demonstrated that LFA-1 engagement in T cells can modulate the activities of signaling proteins, adaptor proteins and enzymes [18, 19, 21, 33]. Lck and ZAP-70 in CD4^+^ T cells play important roles in both the TCR signal-triggering module and LFA-1/ICAM-1 signaling pathway [34–36]. Then, we explored whether the above-observed G-CSF-mediated inhibition of CD4^+^ T cell functions was associated with the activation of Lck and ZAP-70 in LFA-1 signaling. After stimulated with anti-CD3 and ICAM-1, the levels of phosphorylated Lck and ZAP-70 were significantly lower in the G-CSF mobilized CD4^+^ T cells than in the control group CD4^+^ T cells (Figure 5A-5D). In addition, the expressions of total Lck and ZAP-70 were significantly lower in the G-CSF group (Figure 5A and 5B, 5E and 5F). These findings indicated that G-CSF mobilization inhibited LFA-1 signaling by reducing the expression of Lck and ZAP-70, which ultimately inhibited CD4^+^ T cell functions.

### G-CSF stimulation reduced the expression of Lck and ZAP-70 *in vitro*

**Figure 6:**
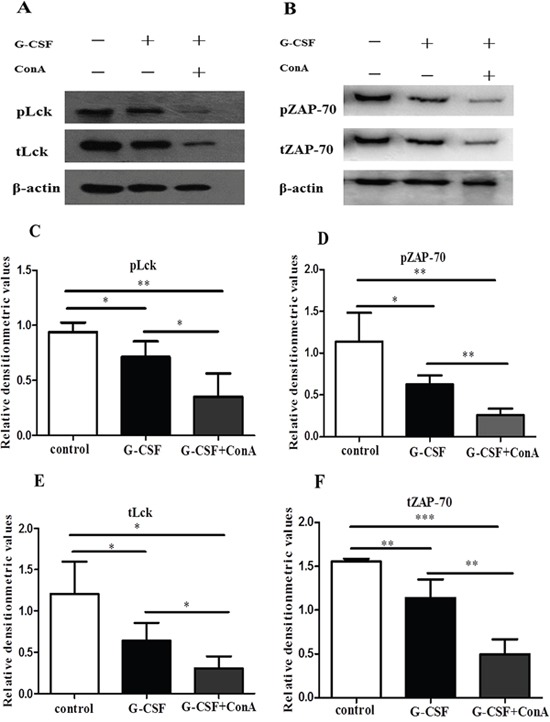
G-CSF stimulation suppressed the expression of Lck and ZAP-70 *in vitro* Purified CD4^+^ T cells were added to 24-well plates and incubated in RPMI alone or in RPMI containing G-CSF or G-CSF plus ConA for 24 h. After the incubation, the cells were transferred to anti-CD3- and ICAM-1-coated 6-well plates and incubated for 1 h at 37°C. **(A** and **B)** Lysates from the three groups of CD4^+^ T cells were analyzed by western blot to detect the expression of Lck, ZAP-70 and phosphorylated Lck and ZAP-70, which were normalized to actin. **(C-F)** Densitometry quantification was performed and presented. All data are represented as the mean ± SD (n=4 per group). * *P* < 0.05; ** *P* < 0.01; *** *P* < 0.001.

Our previous results showed that ConA induced G-CSFR expression in CD4^+^ T cells. To address whether *in vitro* stimulation with G-CSF could directly decrease the expression of Lck and ZAP-70 in LFA-1 signaling, CD4^+^ T cells were unstimulated (control group) or stimulated with 200 ng/ml of G-CSF (G-CSF group) or 200 ng/ml of G-CSF plus 10 ng/ml of ConA (G-CSF+ConA group) for 24 h. After the stimulation, the cells were transferred to anti-CD3- and ICAM-1-coated 6-well plates and incubated for 1 h at 37°C. The results showed that the expressions of Lck and ZAP-70 were significantly lower in the G-CSF stimulated CD4^+^ T cells than in the unstimulated CD4^+^ T cells (Figure 6A-6F). Moreover, the total and phosphorylation levels of Lck and ZAP-70 were significantly decreased in G-CSF+ConA group (Figure 6A-6F). These *in-vitro* results was consistent with the G-CSF mobilization effect above, indicating that G-CSF could inhibit the function of CD4^+^ T cells through down-expression of Lck and ZAP-70 in LFA-1 signaling pathway.

## DISCUSSION

We have previously found that G-CSF decreased the proliferation and activation of CD4^+^ T cells through changing the conformation of LFA-1 [23]. In the present study, we indicated that G-CSF directly suppressed LFA-1-mediated CD4^+^ T cell functions by inhibiting Lck and ZAP-70. These findings indicated that the immunosuppressive effect of G-CSF on T cells through LFA-1/ICAM-1 signaling pathway could be a possible target to reduce GVHD.

To investigate why the incidence aGVHD of PBSCT is no higher than that of conventional BMT, most studies focused on the regulatory effects of G-CSF on donor cells in the grafts [37–40]. In this study, we found that G-CSF mobilization could increase the frequency of lymphocytes in the grafts, which was consistent with presvious studies [27, 39, 41]. Specifically, the frequency of T cells, not B cells and NK cells, was increased after G-CSF mobilization. Currently, there are some controversies about the effects of G-CSF mobilization on the frequencies of NK-cell and B-cell [29, 39, 41–43]. Different G-CSF doses may contribute to the discrepancies in these studies. However, the detailed reasons were unclear. Our results showed that the frequency of CD3^+^CD4^+^ T cells significantly increased in G-CSF-mobilized donors. This observation was in accordance with a previous study [44].

We used MACS kit to isolate CD4^+^ T cells to avoid interference from other cells, and the purity of CD4^+^ T cells was greater than 96%. After ICAM-1 and anti-CD3 activation, G-CSF mobilization decreased the levels of several pro-inflammatory cytokines, including IL-2, TNF-α and IFN-γ, and anti-inflammatory cytokines, such as IL-4 and IL-10. These data are supported by some previous studies [39, 40, 45]. Others studies found that G-CSF increased TH2 cytokine secretion [46, 47]. These discrepancies may due to differences in the sensitivity of the different methods for cytokine detection or to use different reagents *in vitro* cell stimulation. Moreover, flow cytometric analysis indicated that G-CSF mobilization decreased the percentage of TH1 and TH2 cells, but did not alter TH1/TH2 the ratio. The inhibitory effect of G-CSF on cytokine secretion from donor T cells supported the theory that G-CSF attenuated GVHD after PBSC transplantation.

Previous studies had indicated that LFA-1/ICAM-1 signaling was involved in modulating T cell functions, including activation and proliferation [48, 49]. In this study, LFA-1 antibody blockade abrogated TH1 polarization, suggesting the important role of LFA-1 signaling in CD4^+^ T cell function. The LFA-1/ICAM-1 interaction initiates immune responses through the migration of T cells toward inflamed tissues or secondary lymphoid organs. The migration of T cells includes a sequence of rolling, arrest, firm adhesion, polarization and diapedesis [13, 14, 18, 50]. Our results demonstrated that G-CSF inhibited LFA-1-mediated CD4^+^ T cell adhesion, polarization and migration, which may explain previously reported transient defects in the T-cell-mediated immune response [31]. The immunosuppressive effect of G-CSF on T cells may explain why G-CSF mobilized PBSCT has comparable incidence and severity of GVHD with BMT, even though PBSCT contains 10 times more T cells than BMT.

Our *in vitro* results, where purified CD4^+^ T cells were stimulated with G-CSF, supported the idea of direct interaction between G-CSF and G-CSFR-positive T cells. Previous studies also indicated that activated CD4^+^ T and CD8^+^ T cells can express the G-CSFR [31, 32, 46]. Furthermore, Bunse et al. showed that G-CSF greatly affect the activities of cytotoxic CD8^+^ cells and CD4^+^ T cells [51], which indicated that G-CSF may have direct effects on these cells. Src kinases have been shown to play a major role in the G-CSFR signaling pathways [52, 53]. Borneo and colleagues [54] showed that a Src family kinase negatively regulated hematopoietic stem cell mobilization. Lck, a Src kinase, induces Lckdependent phosphorylation of ZAP-70 following TCR triggering, which leads to the phosphorylation of downstream adaptors, up-regulation of integrin affinity signaling molecules and the activation of LFA-1 [35, 36, 55, 56]. We hypothesized that the G-CSF/G-CSFR signaling pathway may first inhibit the expression of the Lck and then down-regulate downstream kinases, adaptors and signaling molecules. Then, this signaling pathway changes the conformation of LFA-1, which ultimately inhibits CD4^+^ T cell functions. Understanding the potential immunosuppressive effect of G-CSF on donor T cells could provide an experimental foundation for the translation of this therapeutic target to clinic. Based on our results, it can be speculated that a target of the kinase Lck may be critical for the immunosuppressive effect of G-CSF on CD4^+^ T cell functions.

In conclusion, our study provides new mechanistic insight about the immunomodulatory effects of G-CSF on CD4^+^ T cell function. Our results indicated that G-CSF directly inhibited LFA-1-mediated CD4^+^ T cell functions by inhibiting Lck and ZAP-70. The immunosuppressive effect of G-CSF mobilization on donor CD4^+^ T cells deepened our understanding about PBSCT. LFA-1/ICMA-1 pathway may become a potential target for GVHD prophylaxis.

## MATERIALS AND METHODS

### Antibodies and reagents

RPMI 1640, FBS, streptomycin and penicillin were obtained from Gibco Life Technologies (Gibco, Carlsbad, CA, USA). Ficoll-Paque PLUS was obtained from GE Healthcare Life Sciences (GE, Pittsburgh, PA, USA). PBS was purchased from Solarbio Life Sciences (Solarbio, Beijing, China). BSA was obtained from Sigma Aldrich (Sigma, St Louis, MO, USA). Magnetic beads conjugated with an anti-CD4 monoclonal antibody were purchases from Miltenyi Biotec (Miltenyi, Cologne, Germany). PerCP-conjugated anti-CD45, PE/FITC-conjugated anti-CD3, PE/PE-Cy5- conjugated anti-CD4, PE-Cy5-conjugated anti-CD8, FITC-conjugated anti-CD19, FITC/PE-Cy5-conjugated anti-CD56, FITC-conjugated anti-IFN-γ, PE-conjugated anti-IL-4, PE-conjugated anti-CD114, the anti-LFA-1 blocking Ab and anti-CD3 monoclonal antibody were obtained from Biolegend (Biolegend, San Diego, CA, USA). The recombinant human ICAM-1/CD54 Fc chimera was from R&D Systems Inc. (RD, Minneapolis, MN, USA). Fluo4-AM and CCK-8 were obtained from Dojindo Laboratories (Dojindo, Osaka, Japan). Human IFN-γ, IL-2, IL-4, IL-10 and TNF-α were obtained from eBioscience (eBioscience, San Diego, CA, USA). Concanavalin A (ConA) was purchased from Santa Cruz Biotechnology (Santa Cruz, Dallas, TX, USA). G-CSF was provided by PLA General Hospital (Kirin Kunpeng, Shanghai, China). Antibodies against ZAP-70 (#3165P) and phospho-ZAP-70 (Y319) (#2701P) were purchased from Cell Signaling Technology Inc. (Cell Signaling Technology, Danvers, MA, USA). Antibodies against Lck (SC-166627), phospho-Lck (Tyr394) (SC-101728) and β-actin (SC-47778) were purchased from Santa Cruz Biotechnology (Santa Cruz, Dallas, TX, USA). Anti-rabbit and anti-mouse IgG were obtained from Santa Cruz Biotechnology (Santa Cruz, Dallas, TX, USA). The enhanced chemiluminescence (ECL) detection reagent was from GE Healthcare Life Sciences (GE, Pittsburgh, PA, USA).

### Sample collection and processing

Peripheral blood was collected from healthy volunteers (normal controls) or G-CSF mobilized donors (10 μg/kg daily for 5 days). Peripheral blood mononuclear cells (PBMCs) were isolated from whole blood by density gradient centrifugation with Ficoll-Paque™. This study was reviewed and approved by the Medical Ethics Committee of PLA General Hospital.

### Isolation and purity of CD4^+^ T cells

Human CD4^+^ T cells were isolated by positive immunomagnetic selection by exposing PBMCs to magnetic beads conjugated with an anti-CD4 monoclonal antibody prior to separation with a magnetic cell sorter (MACS). The isolated CD4^+^ T cells were stained with FITC-conjugated anti-CD3 and PE-conjugated anti-CD4 mAbs. The purity of the stained cells was detected using a flow cytometer. Freshly purified CD4^+^ T cells were then cultured in RPMI 1640 supplemented with 10% FBS and 1% streptomycin and penicillin at 37°C in a 5% CO_2_ atmosphere.

### Lymphocyte and T cell subtype analysis

PBMCs were separated from the whole blood of healthy volunteers and G-CSF- mobilized donors using density gradient centrifugation with Ficoll-Paque™. Lymphocytes subpopulations, including T cells, B cells and NK cells, and T cell subpopulations, including CD3^+^CD4^+^ and CD3^+^CD8^+^ T cells, were stained with the corresponding fluorescently labeled antibodies. The stained cells were detected using a flow cytometer (Becton Dickinson), and the data were analyzed using CXP Analysis 2.1.

### Cytokine measurements

Freshly isolated CD4^+^ T cells from healthy volunteers and G-CSF mobilized donors were stimulated with ICAM-1 or left un-stimulated and then cultured with or without an anti-CD3 mAb (5 μg/ml) or ICAM-1 (3 μg/ml) plus an anti-CD3 mAb (5 μg/ml) for an additional 72 h at 37°C in a 5% CO_2_ incubator. To inhibit LFA-1-mediated signalings, the cells were treated with anti-LFA-1 blocking Ab (100 μg/ml). The levels of IL-2, IFN-γ, TNF-α, IL-4 and IL-10 secreted into the CD4^+^ T cell culture supernatant were measured by the ProcartaPlex™ Multiplex immunoassay according to the manufacturer's instructions.

### Intracellular staining

After a 72 h culture at 37°C in a 5% CO_2_ incubator, in the presence of ICAM-1 (3 μg/ml) and anti-CD3 mAb (5 μg/ml), CD4^+^ T cells from healthy volunteers and G-CSF-mobilized donors labeled with a PE-Cy5-conjugated CD4 mAb and fixed. After membrane permeabilization, the cells were stained with FITC-conjugated anti-IFN-γ and PE-conjugated anti-IL-4 mAbs. After washing, the cells were fixed and analyzed by a flow cytometer.

### Polarization assay

The polarization assay was carried out in 96-well plates as described elsewhere [33, 57, 58]. The 96-well plates were pre-coated overnight at 4°C with 3 μg/ml ICAM-1-Fc and 5 μg/ml anti-CD3 mAb in PBS buffer. Then, the wells were blocked with 2.5% BSA-PBS for 2 h at 37°C and washed twice with RPMI 1640. Next, CD4^+^ T cells (8×10^4^ cells per well) were added to the wells, followed by incubation of the plates for 20 minutes at 37°C. Then, the unbound cells were gently washed off, RPMI 1640 was added to the wells, and the plates were then incubated for another 40 minutes at 37°C. The polarized cells were analyzed by fluorescence microscopy.

### Confocal microscopy

Small round glass confocal dishes 13 mm in diameter were pre-coated with ICAM-1Fc (3 μg/ml) and an anti-CD3 mAb (5 μg/ml) in PBS overnight at 4°C. The small confocal dishes were blocked with 2.5% BSA in PBS for 2 h at 37°C in a 5% CO_2_ atmosphere. The dishes were washed three times with PBS. Then, Fluo4-AM (1 mmol/L) was added to the CD4^+^ T cells, which were then incubated at 37°C in a 5% CO_2_ atmosphere for 30 minutes and then transferred to the coated small confocal dishes and incubated for 20 minutes at 37°C. Finally, the images and track velocity were captured on a Perkin Elmer confocal microscope equipped with a real-time imaging analysis system.

### G-CSFR expression analysis

Purified CD4^+^ T cells (1×10^6^ cells per well) from healthy volunteers were added to 24-well plates and incubated in RPMI alone (control group) or in RPMI containing 200 ng/ml G-CSF (G-CSF group) or 200 ng/ml G-CSF plus 10 μg/ml ConA (G-CSF+ConA group) for 24 h at 37°C in a 5% CO_2_ atmosphere. After the incubation, cells were stained with PE-Cy5-conjugated anti-CD4 and PE-conjugated anti-CD114 mAbs. After washing, the cells were fixed and analyzed by a flow cytometer.

### *In vitro* adhesion assay

Briefly, 96-well plastic dishes were pre-coated as described above. The isolated CD4^+^ T cells (5×10^5^ cells per well) were seeded into 24-well plates with or without 200 ng/ml G-CSF, followed by incubation for 24 h at 37°C in a 5% CO_2_ atmosphere. The cells were then added to the anti-CD3- and ICAM-1-coated wells, and the plates were incubated for 3 h at 37°C. The attachment assay was carried out using a CCK-8 assay kit according to the manufacturer's instructions.

### Western blot analysis

Purified CD4^+^ T cells (1×10^6^ cells per well) from the control group and G-CSF–mobilization group were added to anti-CD3- and ICAM-1-coated 6-well plates and incubated for 1 h at 37°C. For the *in vitro* assay, purified CD4^+^ T cells (1×10^6^ cells per well) from healthy volunteers were added to 24-well plates and incubated in RPMI alone or in RPMI containing 200 ng/ml G-CSF or 200 ng/ml G-CSF plus 10 μg/ml ConA for 24 h. After the incubation, cells were transferred to anti-CD3- and ICAM-1-coated 6-well plates and incubated at 37°C for 1 h. Then CD4^+^ T cells were then harvested and lysed in lysis buffer for 30 minutes on ice. Then, 5×SDS loading buffer was added, and the samples were denatured. Lysates from each sample were separated by 8% SDS-PAGE and transferred to PVDF membranes. The membranes were blocked with 5% non-fat milk and probed overnight at 4°C with the following primary antibodies: anti-ZAP-70 (dilution, 1:1000), anti-Lck (dilution, 1:200), anti-phos-pho-ZAP-70 (dilution, 1:1000), anti-phospho-Lck (dilution, 1:200) and anti-β-actin (dilution, 1:500). Then, the membranes were incubated with horseradish peroxidase-conjugated secondary antibodies (anti-rabbit or anti-mouse IgG, 1:2000) for 45 minutes at room temperature. After washing, the blots were developed using an ECL detection reagent. Densitometric analyses of the western blots were performed using Image J software.

### Statistical analysis

The Data are presented as the means ± standard deviation (SD), and the significance of differences was determined using unpaired and paired Student's t tests (GraphPad Prism software, version 5, USA). Significant differences were indicated as follows: *, *P* < 0.05; **, *P* < 0.01; ***, *P* < 0.001.
